# Shared genetic links between frontotemporal dementia and psychiatric disorders

**DOI:** 10.1186/s12916-022-02335-y

**Published:** 2022-05-05

**Authors:** Chunyu Li, Dejiang Pang, Junyu Lin, Tianmi Yang, Huifang Shang

**Affiliations:** Department of Neurology, Laboratory of Neurodegenerative Disorders, National Clinical Research Center for Geriatric, West China Hospital, Sichuan University, No.37, Guoxue Lane, Chengdu, 610041 Sichuan China

**Keywords:** Frontotemporal dementia, Psychiatric disorders, Genetic correlation, Mendelian randomization

## Abstract

**Background:**

Epidemiological and clinical studies have suggested comorbidity between frontotemporal dementia (FTD) and psychiatric disorders. FTD patients carrying specific mutations were at higher risk for some psychiatric disorders, and vice versa, implying potential shared genetic etiology, which is still less explored.

**Methods:**

We examined the genetic correlation using summary statistics from genome-wide association studies and analyzed their genetic enrichment leveraging the conditional false discovery rate method. Furthermore, we explored the causal association between FTD and psychiatric disorders with Mendelian randomization (MR) analysis.

**Results:**

We identified a significant genetic correlation between FTD and schizophrenia at both genetic and transcriptomic levels. Meanwhile, robust genetic enrichment was observed between FTD and schizophrenia and alcohol use disorder. Seven shared genetic loci were identified, which were mainly involved in interleukin-induced signaling, synaptic vesicle, and brain-derived neurotrophic factor signaling pathways. By integrating cis-expression quantitative trait loci analysis, we identified *MAPT* and *CADM2* as shared risk genes. MR analysis showed mutual causation between FTD and schizophrenia with nominal association.

**Conclusions:**

Our findings provide evidence of shared etiology between FTD and schizophrenia and indicate potential common molecular mechanisms contributing to the overlapping pathophysiological and clinical characteristics. Our results also demonstrate the essential role of autoimmunity in these diseases. These findings provide a better understanding of the pleiotropy between FTD and psychiatric disorders and have implications for therapeutic trials.

**Graphical abstract:**

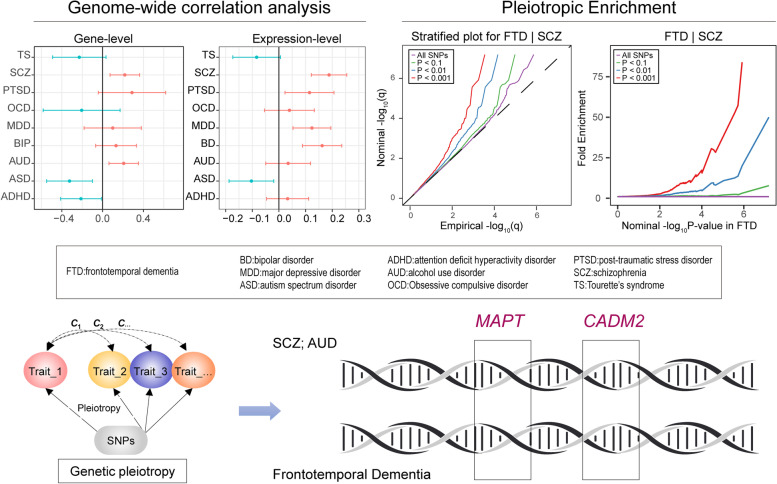

**Supplementary Information:**

The online version contains supplementary material available at 10.1186/s12916-022-02335-y.

## Background

Frontotemporal dementia (FTD) is a clinically and pathologically heterogeneous group of non-Alzheimer dementias, characterized by progressive deficits in behavior, executive function, or language [1]. Despite the diminished quality of life for the patients with FTD, substantial costs, and caregiver burden [2], little progress has been made in the development of effective cures for FTD. Current available symptomatic treatments only provide limited clinical utility [3]. Therefore, exploring the pathogenesis of FTD and developing novel therapeutic strategies are necessary and urgent to reduce this huge socioeconomic burden.

The clinical symptoms of FTD are heterogeneous, with more than 30% of patients manifesting psychotic symptoms [4]. The early symptoms of behavioral variant FTD (bvFTD) overlap considerably with common primary psychiatric disorders like schizophrenia and major depressive disorder (MDD) [5, 6]. For example, psychotic symptoms like psychosis and hallucinations, which are characteristic of schizophrenia, are frequently reported in patients with FTD [4, 7], while cognitive impairments such as dysfunction of working memory and verbal learning have been documented extensively in schizophrenia [8]. MDD and bvFTD share common symptoms like lack of interest, decreased motivation, and impaired concentration [5]. Meanwhile, potential shared molecular pathophysiology has been proposed between psychiatric disorders and FTD like brain derived neurotrophic factor (BDNF) and progranulin [9]. In addition, relatives of patients carrying *C9orf72* mutations, a common genetic cause for FTD, were in higher probability to develop schizophrenia, late-onset psychosis, suicide, and autism spectrum disorder [10]. These associations raise the possibility of shared genetic or environmental risk factors between FTD and psychiatric disorders, which is still mostly unexplored. Therefore, a systematic analysis is necessary to decipher whether shared pleiotropic risk variants exist between FTD and psychiatric disorders and whether specific molecular biological pathways are involved.

Growing studies have suggested that complex diseases often have a highly polygenic structure, with amounts of genetic variants with small effects contributing to the risk of the disease, like FTD. However, due to the limited sample size in the genome-wide association studies (GWAS) of FTD so far (*N* = 6462), the variants with relatively smaller effect size could not be identified. Recently, a genetic pleiotropic conditional false discovery rate (FDR) approach was developed to investigate genetic overlap between polygenic traits using summary data from GWAS and has been utilized extensively in several human traits and diseases [11–13]. By integrating GWAS results from multiple phenotypes, this method could provide insights into the genetic pleiotropy and increased statistical power to discover less significant associations [12–14]. Applying this approach, we systematically evaluated the shared genetic background between FTD and psychiatric disorders and further conducted functional enrichment analysis and Mendelian randomization (MR) analysis, as was shown in the graphical abstract.

## Methods

### GWAS summary statistics

We investigated the genetic links between FTD (*N* = 6,462) [15] and nine psychiatric disorders including attention deficit hyperactivity disorder (ADHD) (*N* = 55,374) [16], autism spectrum disorder (ASD) (*N* = 46,350) [17], alcohol use disorder (AUD) (*N* = 121,604) [18], bipolar disorder (BD) (*N* = 45,871) [19], major depressive disorder (MDD) (*N* = 500,199) [20], obsessive-compulsive disorder (OCD) (*N* = 9,725) [21], post-traumatic stress disorder (PTSD) (*N* = 206,655) [22], schizophrenia (SCZ) (*N* = 77,096) [23] and Tourette’s syndrome (TS) (*N* = 14,307) [24] based on summary statistics from previous GWAS. The FTD GWAS is a meta-analysis of four subtypes including bvFTD, semantic dementia, progressive non-fluent aphasia (PNFA), and FTD overlapping with motor neuron disease (MND), covering the most relevant FTD clinical signatures. Considering that each subtype of FTD might be genetically heterogeneous, we further analyzed summary statistics from the four subtypes of FTD [15]. Details of the summary data for the utilized GWAS were shown in Additional file 1: Table S1. The study design including the diagnosis criteria, collection of samples, quality control procedures, and imputation methods have been described in each publication. The research protocol of each GWAS was approved by the relevant institutional review boards or ethics committees.

### Statistical analyses

#### Genetic correlation

**Fig. 1:**
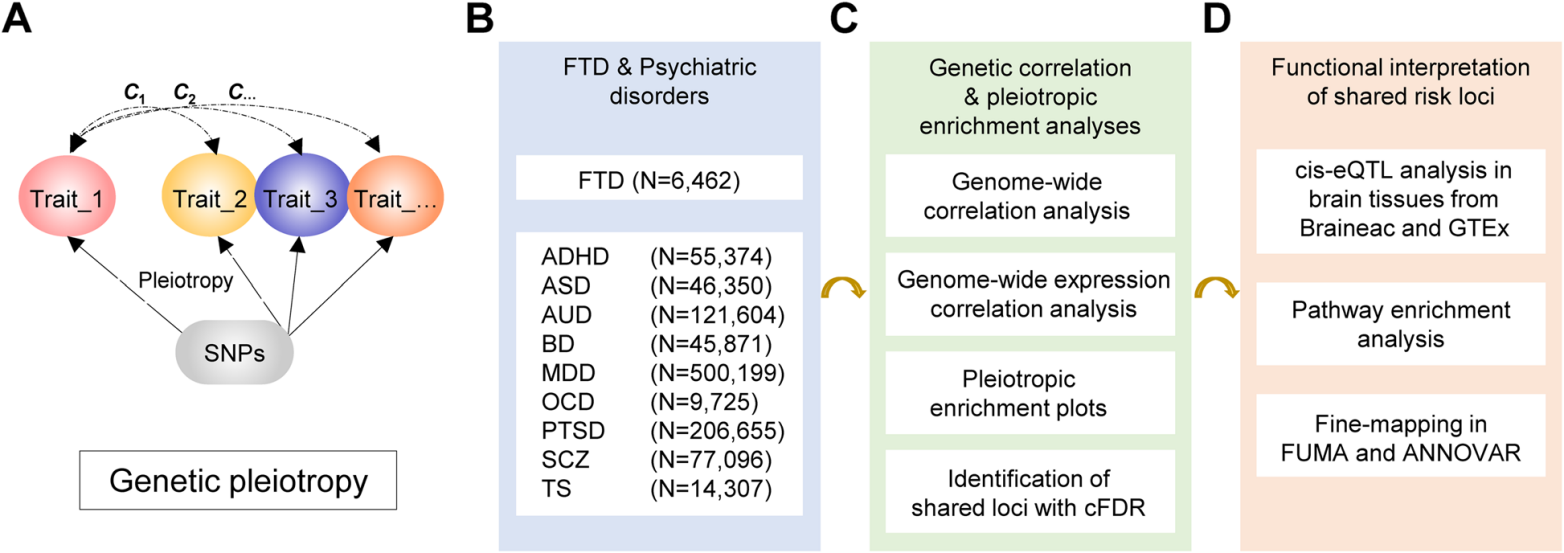
Schematic overview of the analytical workflow. **A** Illustration of genetic pleiotropy. **B** Analyzed diseases. **C** Analytical workflow. **D** Functional interpretation of the results. FTD, frontotemporal dementia; BD, bipolar disorder; MDD, major depressive disorder; ASD, autism spectrum disorder; ADHD, attention deficit hyperactivity disorder; AUD, alcohol use disorder; OCD, Obsessive compulsive disorder; PTSD, post-traumatic stress disorder; SCZ, schizophrenia; TS, Tourette’s syndrome

The schematic overview of the analytical workflow was shown in Fig. 1. We firstly estimated the genetic correlation between each psychiatric disorder and FTD as well as its subtypes using GNOVA [25]. GNOVA estimates genetic covariance with summary data of the genetic variants shared between two GWAS and then calculates the genetic correlation based on genetic covariance and variant-based heritability. We ran GNOVA on single nucleotide polymorphisms (SNP) in both diseases together with reference data derived from the 1000 Genomes Project European population using default parameters. For each dataset, we applied the same quality-control steps described in Bulik-Sullivan et al. [26] using the munge_sumstats.py (included in GNOVA). The major histocompatibility complex (MHC) region, defined as base positions from 24,000,000 to 35,000,000 on chromosome 6 (GRCh37), was excluded from the analysis due to its complex linkage disequilibrium (LD) structure. A *P* value below 0.006 (0.05/9) was considered significant after the Bonferroni correction.

#### Gene expression overlap

We further investigated whether the genetic overlap between FTD and psychiatric disorders was mediated by shared regulation of gene expression. We generated tissue-specific, disease-inferred gene expression profiles using TWAS software with default parameters. TWAS integrates gene expression measurements with GWAS summary statistics to identify genes whose cis-regulated expression is associated with complex traits [27]. Since both FTD and psychiatric disorders were neurological disorders with clinical complications mainly in the brain, we analyzed RNA-seq data from the brain and whole blood tissues in the GTEx v7 reference panel. Then, we estimated the overlap between the disease-inferred gene expression with RHOGE [28] using TWAS results with nominal association (*P* < 0.05). RHOGE estimates the correlation between two traits which can be attributed to cis-expression quantitative trait loci (eQTL) as represented by different trait-inferred gene expression profiles. The MHC was excluded from the analysis due to its complex LD structure. A *P* value below 0.006 (0.05/9) was considered significant after the Bonferroni correction.

#### Genomic control

Due to population stratification or cryptic relatedness or overcorrection of test statistics [29], the empirical null distribution in GWAS is sometimes inflated or deflated [11–13]. To correct such bias, we applied a genomic control method leveraging intergenic SNPs to adjust the summary statistics for each GWAS respectively (Additional file 2). Then, we pruned the SNPs by removing SNPs in LD (*r*^2^ > 0.2 within 250 kb) based on 1000 Genomes Project LD structure using plink clump functionality [30].

#### Pleiotropic enrichment plots

To assess the pleiotropic enrichment, we plotted conditional quantile-quantile plots for FTD by creating subsets of SNPs based on their associations with each psychiatric disorder, and vice versa. To further quantitatively assess the level of enrichment, we constructed fold-enrichment plots of nominal -log_10_(*P*) values of FTD for all SNPs and subsets of SNPs determined by the significance of the association with each psychiatric disorder [11–13], and vice versa (Additional file 2).

#### Identification of risk loci

To identify risk loci associated with FTD and its subtypes conditional on each psychiatric disorder, we computed the conditional FDR statistics using the conditional FDR approach [11–13] (Additional file 2). Briefly, the FDR method is based on Bayesian statistics, and the conditional FDR is the posterior probability that a given SNP is null for the first phenotype given that the *P* values for both phenotypes are as small as or smaller than the observed *P* values. Furthermore, to identify shared risk loci between FTD and each psychiatric disorder, we computed the conjunctional FDR statistics. To reduce false positives, a strict significance threshold of FDR < 0.01 was utilized, corresponding to one false positive per 100 reported associations. We defined independent genomic loci using FUMA with default parameters [31], which is an online platform for functional annotation and interpretation of genetic variants (http://fuma.ctglab.nl/). LD information was calculated using reference data of the European population from the 1,000 Genomes Project.

We next conducted gene-based association analysis using MAGMA (Multi-marker Analysis of GenoMic Annotation) with default parameters to integrate association signals from the SNP level into gene level [32]. Genes with a significant association in both FTD or its subtypes and psychiatric disorders were considered as shared risk genes. The European-ancestry subjects from the 1000 Genomes Project (Phase 3) were used for the LD reference. *P* value was adjusted by Bonferroni correction according to the number of tested genes.

#### Functional evaluation of shared risk loci

To assess whether the shared risk loci modify gene expression, we evaluated cis-eQTL in Braineac, a publicly available dataset of normal control brains for investigating the genes and SNPs associated with neurological disorders [33]. We analyzed eQTL across ten brain regions including the cerebellum, frontal cortex, hippocampus, medulla, occipital cortex, putamen, substantia nigra, temporal cortex, thalamus, and white matter. To minimize false positives, a *P* value below 1.7E−07 (0.05/292,000 probes) was considered significant after the Bonferroni correction. Meanwhile, we analyzed cis-eQTL in 13 brain tissues from GTEx v7 [34] (amygdala, anterior cingulate cortex (BA24), caudate basal ganglia, cerebellar hemisphere, cerebellum, cortex, frontal cortex (BA9), hippocampus, hypothalamus, nucleus accumbens basal ganglia, putamen basal ganglia, spinal cord cervical, substantia nigra). Cis-eQTLs as pre-computed by GTEx were downloaded directly from the GTEx portal (http://gtexportal.org/). We applied a *P* value cutoff of 1E−06 to identify significant cis-eQTLs, which approximates a false discovery threshold of 0.05 as suggested by previous analysis [35].

To identify enrichment in gene ontologic features associated with FTD and psychiatric disorders, we used ConsensusPathDB [36] for functional interaction analysis. The shared risk genes identified with the conjunctional FDR method and eQTL analyses were utilized with default parameters and background gene sets. Biological, cellular, and molecular gene ontologic terms were analyzed. Genes in the MHC region were excluded due to the complex LD patterns. A *P* value below 0.05 was considered significant after correcting for multiple testing using the FDR method.

#### Mendelian randomization analysis

To evaluate the causative effect of psychiatric disorders on the risk of FTD, we performed a two-sample MR analysis using the random effects inverse variance weighted (IVW) method [37]. Single nucleotide polymorphisms (SNP) that passed the genome-wide significance threshold (*P* < 5E−08) were chosen as instrument variables, which were then clumped based on the 1000 Genomes Project LD structure. Given that only one or no locus was significant for ASD, OCD, PTSD, and TS, a more relaxed significance threshold (*P* < 1E−06) was used. Index SNPs (*R*^2^ < 0.001 with any other associated SNP within 10 Mb) with the minimum *P* value were kept. Harmonization was undertaken to rule out strand mismatches and ensure alignment of SNP effect sizes. A *P* value below 0.006 (0.05/9) was considered statistically significant after the Bonferroni correction.

In the second stage, we evaluated whether FTD as a risk factor could causally influence the risk of psychiatric disorders and performed the MR analysis using the same workflow. Given that only one locus was significant for FTD, a more relaxed significance threshold (*P* < 1E−06) was used. Since the sample size of the GWAS for each subtype of FTD was small and few significant SNPs were identified, we did not analyze each subtype of FTD as a risk factor.

In addition, we conducted sensitivity analyses to estimate potential violations of the model assumptions in the MR analysis. We conducted Mendelian randomization pleiotropy residual sum and outlier (MR-PRESSO) analysis and leave-one-out analysis to detect outlier instrumental variables [38]. Outlier instrumental variables identified by the MR-PRESSO outlier test were removed step-by-step to reduce the effect of horizontal pleiotropy. Cochran’s *Q* test was executed to check the heterogeneity across the individual causal effects. MR-Egger regression was performed to evaluate the directional pleiotropy of instrumental variables [39]. To evaluate the strongness of each instrumental variable, we computed the F-statistic of each SNP [40]. The statistical power was calculated using an online tool at http://cnsgenomics.com/shiny/mRnd/ [41]. The statistical analyses were conducted using the R package TwoSampleMR 0.5.5 [42].

## Results

### Genetic correlation

**Fig. 2:**
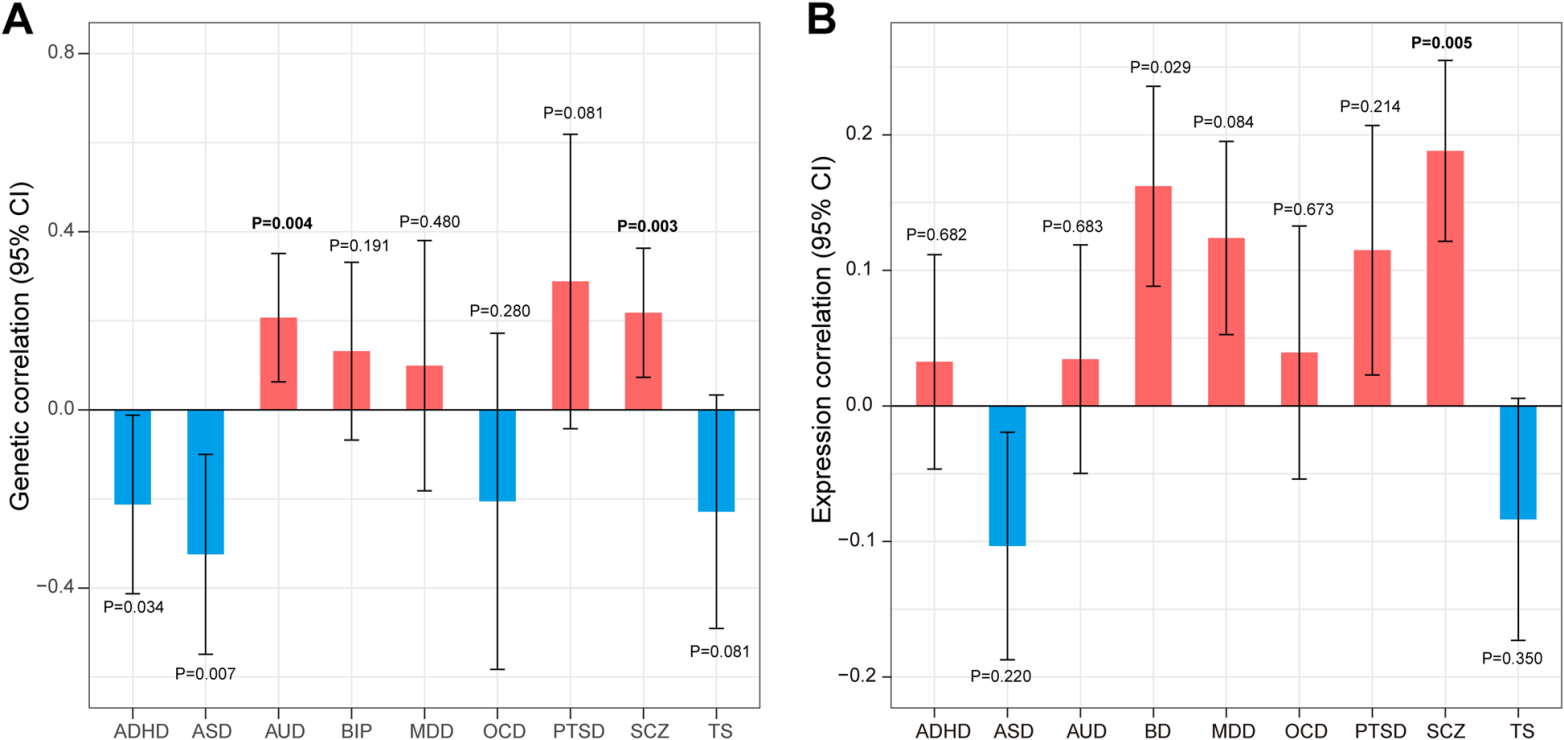
Correlation between FTD and psychiatric diseases. **A** Genetic correlation. **B** Expression correlation. Error bars indicate 95% confidence intervals. Red color indicates positive correlation, while blue color indicates negative correlation. Bold *P* value denotes significance after the Bonferroni correction

We first estimated the genetic correlation between FTD and each psychiatric disorder. We identified a significant positive genetic correlation between FTD and SCZ and AUD (Fig. 2A). At transcriptomic level, we identified a significant positive correlation between FTD and SCZ (expression correlation: 0.19; 95% CI: 0.12–0.26; *P* = 5.22E−03) (Fig. 2B). From the plot, we can also see that the direction of genetic correlation and expression correlation were mostly consistent. In the subtype analyses, a significant genetic correlation was identified between bvFTD and SCZ, AUD, as well as FTD_MND and SCZ (Additional file 1: Fig. S1).

### Estimation of pleiotropic enrichment

**Fig. 3:**
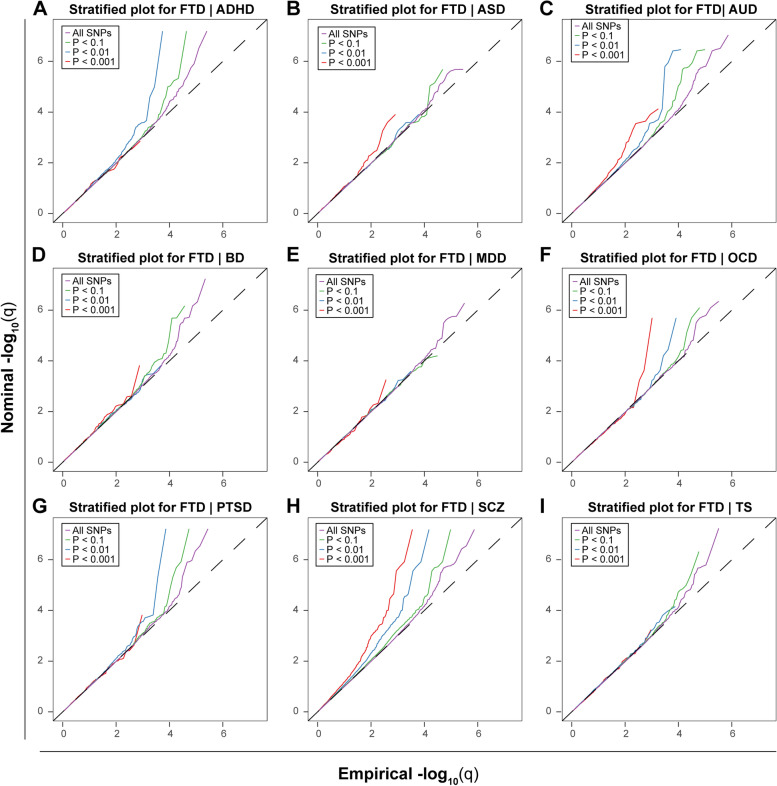
Enrichment plots. Conditional quantile-quantile plots of nominal versus empirical -log_10_(*P*) of FTD as a function of significance of association with psychiatric diseases at the levels of -log_10_(*P*) > 0, -log_10_(*P*) > 1, -log_10_(*P*) > 2, and -log_10_(*P*) > 3, which correspond to *P* < 1, *P* < 0.1, *P* < 0.01, and *P* < 0.001, respectively. Dotted lines indicate the expected line under the null hypothesis, and leftward deflection demonstrates degree of enrichment

**Fig. 4:**
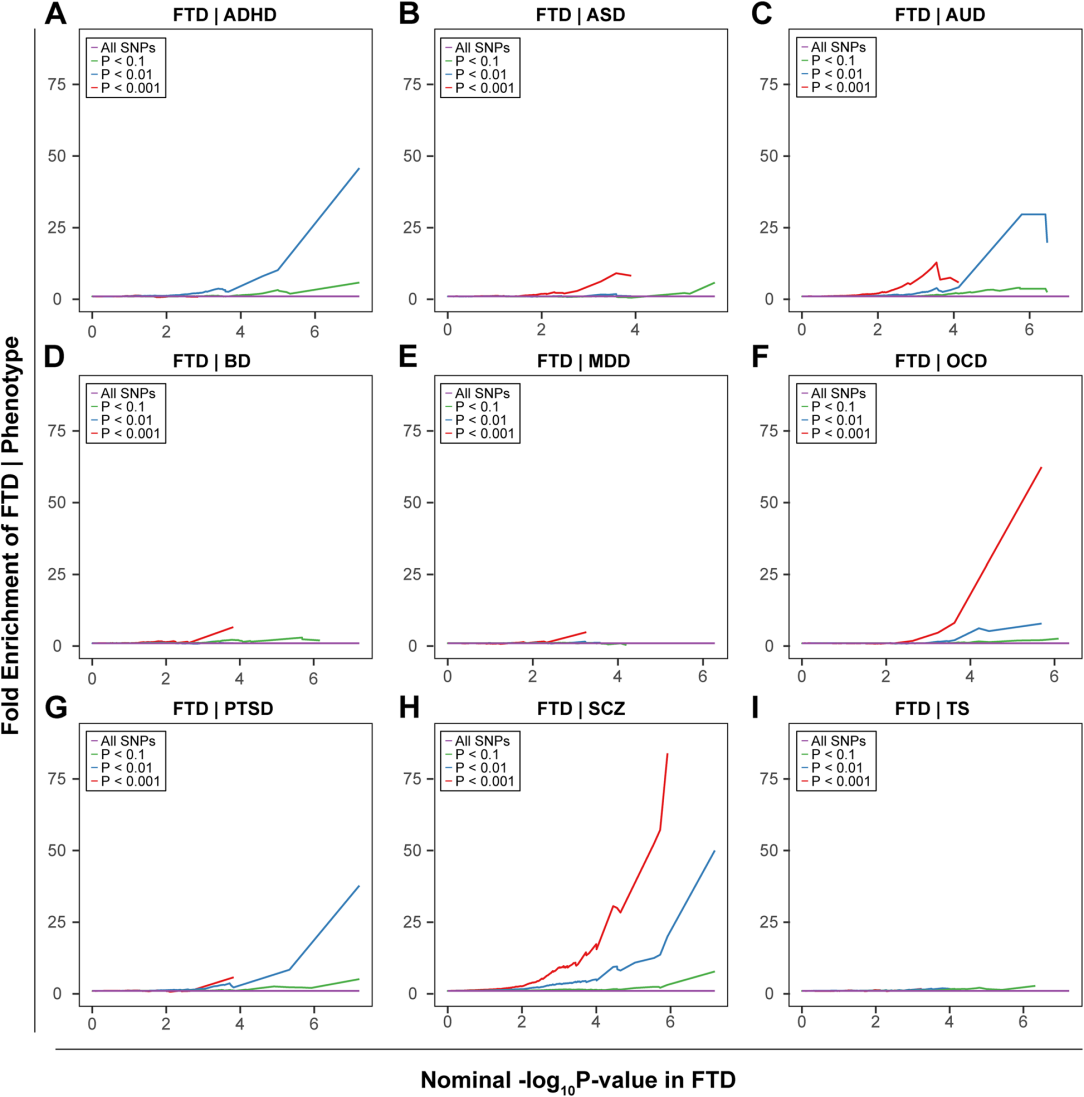
Fold-enrichment plots. Enrichment plots of nominal -log_10_(*P*) of FTD as a function of significance of association with psychiatric diseases at the levels of -log_10_(*P*) ≥ 0, -log_10_(*P*) ≥ 1, -log_10_(*P*) ≥ 2, and -log_10_(*P*) ≥ 3, which correspond to *P* ≤ 1, *P* ≤ 0.1, *P* ≤ 0.01, and *P* ≤ 0.001, respectively. The horizontal lines indicate the expected line under the null hypothesis, and leftward deflection demonstrates degree of enrichment

Successive enrichment was observed in the stratified quantile-quantile plots for FTD conditional on association *P* values with SCZ, AUD, and OCD, indicating that the proportion of non-null SNPs in FTD increased with higher levels of association with these three psychiatric disorders. Partial enrichment for PTSD and ADHD was observed since the QQ plot for SNPs with *P* ≤ 1E−03 was not deflected further (Fig. 3). From the opposite direction, successive enrichment was observed in the stratified quantile-quantile plots for SCZ, AUD, and ASD as a function of FTD (Additional file 1: Fig. S2). In the fold-enrichment plots, we could observe over 200-fold enrichment for FTD conditional on SCZ, approximately 60-fold enrichment conditional on OCD, 45-fold enrichment conditional on ADHD, 35-fold enrichment conditional on PTSD, and 30-fold enrichment conditional on AUD (Fig. 4). From the opposite direction, we could observe over 75-fold enrichment for SCZ conditional on FTD, approximately 70-fold enrichment for AUD and 25-fold enrichment for ASD (Additional file 1: Fig. S3).

### Risk loci identified with conditional FDR

To discover genetic variants associated with FTD conditional on each psychiatric disorder, we performed the conditional FDR statistical analysis. A total of 13 risk loci were identified with conditional FDR < 0.01 (Additional file 1: Table S2). Seventeen SNPs were not suggestively significant (*P* < 1.0E−06) in the original GWAS of FTD. Among the identified risk genes, *MAPT* is an established causative gene for FTD, and several genes like *BTNL2* and *HLA-DRA* in the MHC region have been reported to be closely related to FTD. Due to the complex LD pattern in the MHC region, we estimated the LD between newly identified loci in the MHC region (rs3132451, rs3130291, rs60045856) and significant loci in the original FTD GWAS using the 1000 Genomes Project data. As a result, no variant was in strong LD (*r*^2^ > 0.8) with the three newly identified variants, suggesting they were independent signals. Meanwhile, six genes near *MAPT* in chromosome 17 including *MAPT-AS1*, *SPPL2C*, *CRHR1*, *KANSL1*, *NSF*, and *WNT3* have been identified as risk genes by earlier GWAS for Parkinson’s disease, another common neurodegenerative disorder which might present clinical symptoms of FTD. To further identify shared loci between FTD and psychiatric disorders, we calculated the conjunctional FDR statistics. A total of seven shared risk loci with conjunctional FDR < 0.01 were identified (Table 1).

**Table 1:** Shared risk loci between FTD and psychiatric disorders

Index SNP	Genomic position	Closest gene	FDR value	Associated phenotype
rs10863728	1:209056568	LINC01717; LINC01774	5.97E-03	ASD
rs10889502	1:65379982	JAK1	4.64E-03	AUD
rs9812061	3:85013262	CADM2	7.38E-03	AUD
rs3132451	6:31582025	AIF1	6.99E-03	SCZ
rs3130291	6:32175331	NOTCH4	5.38E-03	SCZ
rs3117097	6:32358689	HCG23;TSBP1-AS1	3.43E-05	PTSD, SCZ
rs3129953	6:32361821	BTNL2	1.25E-04	PTSD, SCZ
rs7746751	6:32430867	HLA-DRA;HLA-DRB5	9.91E-06	SCZ
rs60045856	6:32799845	TAP2	9.82E-03	SCZ
rs215034	16:15996556	FOPNL;ABCC1	3.10E-03	AUD
rs34104358	17:43508616	ARHGAP27	1.65E-03	ASD,AUD
rs56314414	17:43536970	PLEKHM1	1.52E-03	ASD,AUD
rs62057112	17:43897202	CRHR1; LINC02210-CRHR1	8.50E-04	ASD,AUD
rs62054815	17:43923266	SPPL2C	9.27E-04	ASD,AUD
rs34097347	17:43949448	MAPT-AS1	8.66E-04	ASD,AUD,SCZ
rs62063281	17:44038785	MAPT	8.61E-04	ASD,AUD,SCZ
rs62063675	17:44126575	KANSL1	1.10E-03	ASD,AUD,SCZ
rs199531	17:44830414	NSF	2.18E-03	ASD,AUD,SCZ
rs199526	17:44847707	WNT3	1.40E-03	ASD,AUD
rs1406857	20:37362432	SLC32A1;ACTR5	4.21E-03	SCZ

*SNP* single nucleotide polymorphism, *FDR* false discovery rate, *SCZ* schizophrenia, *ASD* autism spectrum disorder, *AUD* alcohol use disorder, *PTSD* post-traumatic stress disorder. Index SNP was the one with lowest FDR value. Index SNP was the SNP with the lowest FDR value in each locus. The genomic position was on GRCh37. Closest gene was annotated from ANNOVAR

Then, we conducted the conditional FDR analysis for each subtype of FTD conditional on psychiatric disorders. Three loci were identified for bvFTD, including rs17652337 in chromosome 17 and rs9268887 in the MHC region which were identified in the cFDR analysis for FTD as well (Additional file 1: Table S3). The locus rs74977128 (MIR3166;CTSC) was newly identified. Additionally, another locus rs7267772 (NKX2-2;LINC01727) was identified for FTD_SD conditional on ASD. Furthermore, we conducted the conjunctional FDR analysis for each subtype of FTD. As a result, three risk loci were identified for bvFTD and one locus was identified for FTD_SD (Additional file 1: Table S4). No risk loci were identified for the other two subtypes of FTD.

In the results from gene-based association analysis, three genes were significant for FTD, namely *APOE* (*P* = 5.48E−08), *WNT3* (*P* = 2.57E−06), and *TOMM40* (*P* = 2.75E−06). For *APOE* and *TOMM40* which were risk genes for Alzheimer’s disease, no significant association was identified in psychiatric disorders. For *WNT3*, a significant association was identified for AUD (1.17E−10) and ASD (8,17E−08), which was consistent with the results from the cFDR analysis. No significant association was identified for the subtypes of FTD.

### Functional interpretation of shared risk loci

To determine whether the shared risk loci modify gene expression, we evaluated cis-eQTL in brain-related tissues in Braineac and GTEx. As a result, the pleiotropic risk loci affect the expression of *LRRC37A2*, *MAPT*, and *CADM2* in brain-related tissues based on summarized results from both Braineac and GTEx (Additional file 1: Table S5).

Furthermore, to determine whether the identified shared risk genes were involved in specific biological pathways, we conducted pathway over-representation analysis. The shared genes were mainly enriched in the interleukin signaling, synaptic vesicle and BDNF signaling pathways (Additional file 1: Table S6). Additionally, the shared genes were also enriched in 21 GO sets (Additional file 1: Table S7).

### Mendelian randomization analysis

**Fig. 5:**
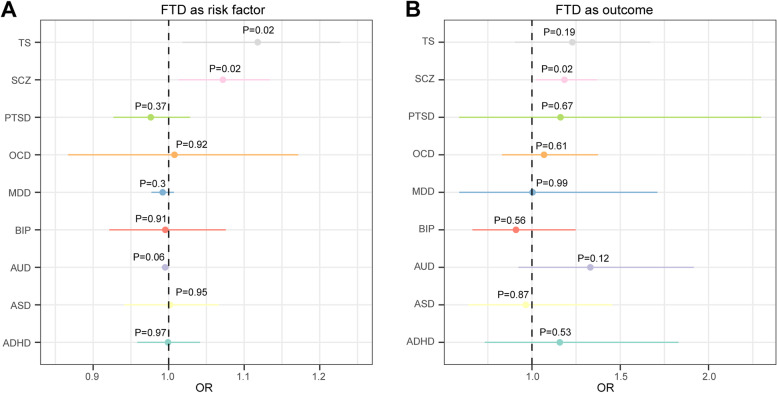
Forest plot showing results from the Mendelian randomization analysis to evaluate potential causal association between FTD and psychiatric disorders. **A** Mendelian randomization analysis results with FTD as risk factor and psychiatric disorders as outcomes. **B** Mendelian randomization analysis results with psychiatric disorders as risk factors and FTD as outcome

Furthermore, we analyzed the role of FTD in the risk of psychiatric disorders, and vice versa. No significant association was identified after the Bonferroni correction (Fig. 5). Notably, FTD was nominally associated with a higher risk of SCZ (OR: 1.07, 95% CI: 1.01–1.13, *P* = 0.017), and SCZ was also nominally associated with a higher risk of FTD (OR: 1.18, 95% CI: 1.02–1.37, *P* = 0.023) (Fig. 5, Additional file 1: Fig. S4). Next, we performed extensive sensitivity analyses to validate the causal association between FTD and psychiatric disorders. The Cochran’s *Q* test did not detect heterogeneity of effects across the instrumental variables (Additional file 1: Table S8). The F statistics of all the instrument variables were above 10 (ranging from 20 to 139), indicating the absence of weakness in the selected instruments. No apparent horizontal pleiotropy was observed as the intercept of MR-Egger was not significantly deviated from zero (Additional file 1: Table S8). Meanwhile, no potential instrumental outlier was detected at the nominal significance level of 0.05 by the MR-PRESSO analysis (Additional file 1: Table S8). The leave-one-out results suggest that the causal effect was not driven by a single instrumental variable (Additional file 1: Fig. S5).

## Discussion

In the current study, we identified significant genetic overlap between FTD and SCZ, AUD. Besides, we identified 13 risk loci for FTD, as well as seven shared loci between FTD and psychiatric disorders, and identified *MAPT* and *CADM2* as shared risk genes by integrating cis-eQTL analysis. Meanwhile, MR analysis suggested mutual causation between FTD and SCZ with nominal association. These results suggested shared genetic architecture and potential common pathogenesis between FTD and psychiatric disorders, provided a better understanding for the pleiotropy of FTD, and had implications in the clinical treatment of related complex phenotypes.

We first estimated the genetic correlation between FTD and psychiatric disorders, and identified a significant positive genetic correlation between FTD and SCZ and AUD. This was consistent with previous observational studies which found comorbidity between FTD and SCZ and AUD [43, 44]. In the subtype analysis, a significant positive genetic correlation was identified between bvFTD and AUD and SCZ, which was similar to the results of FTD. This is not surprising since the majority of patients in the FTD GWAS were patients with bvFTD. Meanwhile, a significant positive genetic correlation was identified between FTD_MND and SCZ. Similarly, previous research has demonstrated a significant genetic correlation between SCZ and amyotrophic lateral sclerosis, a severe motor neuron disease [45]. Additionally, a negative genetic correlation was identified between FTD and some psychiatric disorders like ASD. However, the results should be interpreted with caution since the correlation was not significant. Meanwhile, sample size of some diseases like OCD and TS were relatively small, which decreased the accuracy of the estimated correlation. Further analysis based on GWAS with larger sample size was still necessary.

Substantial genetic overlap was observed between FTD and SCZ. SCZ is a severe psychiatric disorder with characteristic symptoms including delusions and hallucinations [46]. SCZ was originally regarded as a neurodevelopmental disorder [47], in which alterations occurring in prenatal-to-early adolescent development contribute to the pathogenesis. However, the neurodegenerative hypothesis of SCZ has been the subject of discussion since Kraepelin’s observation of mental decline in SCZ patients [48–50], though such hypothesis was still debated due to various factors such as the absence of neurodegenerative characteristics in SCZ like gliosis [51]. Previous studies have identified shared genetic architecture between SCZ and other neurodegenerative disorders including PD [52] and ALS [45] and identified genetic enrichment and shared risk loci using similar methods. Our study identified a significant correlation between FTD and SCZ from both genetic and transcriptive perspectives, supplementing current knowledge about the correlation between SCZ and neurodegenerative disorders [53]. In addition, using the MR approach, we identified shared causation between FTD and SCZ, which further suggested potential shared etiology between these two disorders [43]. Previous research on SCZ has demonstrated changes in the frontal and temporal lobes [54, 55], while FTD is a disorder of frontal lobe function. Meanwhile, previous study identified significantly higher morbid risk for SCZ and schizoaffective disorder in relatives of FTD probands [56]. And patients with SCZ were with a significantly higher risk of developing dementia [53]. These multiple links suggested the potential shared pathogenesis or genetic background between FTD and SCZ, which is worth further exploration. However, the causation should be interpreted with caution since the association was nominally significant. Considering that the sample size of FTD was relatively small, replication using summary statistics from future GWAS with larger sample size was still necessary.

We identified a significant positive genetic correlation between FTD and AUD and observed a substantial genetic enrichment for FTD as a function of AUD. AUD is a medical condition characterized by the inability to stop or control alcohol use despite adverse social, occupational, or health consequences [57]. Alcohol use has close relation with the risk of dementia. Previous epidemiologic studies have shown that consumers of moderate amounts of alcohol might have a decreased risk of dementia and cognitive decline compared with nondrinkers [58, 59], though substantial heterogeneity still exists [59, 60]. In contrast, heavy drinking might increase the risk of dementia [61]. Our study identified a significant correlation between FTD and AUD, consistent with previous nationwide retrospective cohort study which identified AUD as a major risk factor for all types of dementia in around 31 million individuals [62]. Pathologically, chronic and heavy alcohol use could disrupt the availability of nutrients like thiamine that is needed by the brain or directly cause neuronal toxicity. Ethanol and its metabolite acetaldehyde also have a direct neurotoxic effect, leading to permanent structural and functional brain damage. Therefore, heavy drinking was correlated with FTD and should be considered as a risk factor in future clinical investigations of FTD.

We identified multiple shared risk loci in the MHC region for FTD and psychiatric disorders, suggesting the essential role of autoimmunity in these diseases. Immune-mediated dysfunction and neuroinflammation have been shown to play an important role in the pathogenesis of FTD, like cortical inflammation, microglial activation, and astrogliosis [63]. Meanwhile, increased prevalence of immune-mediated diseases was observed among patients with FTD [64, 65]. As for psychiatric disorders, immunological pathways also play an important role in the etiology [66]. A family history of autoimmune diseases was shown to be associated with an increased risk of psychotic disorders and vice versa. Meanwhile, neurodegenerative disorders including FTD and psychiatric diseases are both associated with chronic or flaring inflammation of specific brain areas with infiltration of peripheral immune cells, resulting in mild or severe brain damage that leads to the development of the characteristic disease symptoms [67]. However, considering the complexity of the autoimmunity in the MHC region, it is still elusive how this region was involved in the pathogenesis. The microbiota-gut-brain axis composed of endocrinological, immunological, and neural mediators has been proposed due to its involvement in neurodegenerative and psychiatric disorders [68, 69]. Further exploration is still necessary to understand how the immune system interacts with neurodegenerative and psychiatric disorders and help better understand the shared pathogenesis.

By integrating cis-eQTL data from Braineac and GTEx, we identified two shared risk genes, namely *MAPT* and *CADM2*. *MAPT* encodes the microtubule-associated protein tau, a protein central to Alzheimer’s disease neuropathology. *MAPT* is a well-established causative gene for FTD, and more than 50 *MAPT* mutations have been identified to lead to FTD [70], though no GWAS had linked common variants in *MAPT* to FTD. In addition, FTD patients with *MAPT* mutations showed clinical symptoms that overlap with SCZ [71, 72], suggesting *MAPT* might be related to the pathogenesis of both FTD and SCZ. *CADM2* is a member of the *CADM* family and is highly expressed in the brain tissues. Multiple GWAS have identified that *CADM2* was associated with the cognitive ability [73, 74], suggesting a potential role of *CADM2* in dementia. Meanwhile, *CADM2* was also shown to play a role in some psychiatric disorders like AUD [18], BD [19], and ADHD [75]. Therefore, *CADM2* might act as a potential genetic link between FTD and psychiatric disorders. Notably, since we analyzed cis-eQTL in brain regions only, some eQTLs in other tissues might be missed. With better understanding of the involved tissues for FTD and psychiatric disorders, further exploration in these tissues was still necessary.

Using the pleiotropic genes for functional enrichment analysis in ConsensusPathDB, we identified several enriched pathways, all of which were somehow involved in the pathogenesis of neurological and psychiatric disorders. The cytokine interleukin-6 (IL-6) is a soluble mediator with a pleiotropic effect on inflammation, immune response, and hematopoiesis. The IL-6 influences the physiological homeostasis of neural tissue. Profound neuropathological changes in PD, AD, and FTD are potentially associated with increased IL-6 expression in brain [76, 77]. Meanwhile, IL-6 plays a critical role in psychiatric disorders like MDD and SCZ [78, 79]. Synaptic vesicle proteins modulate the release of neurotransmitters in the synaptic cleft via regulation of vesicle transport, membrane fusion, and exocytosis. Mounting evidence has suggested the important role of synaptic vesicle proteins in the pathophysiology of several psychiatric disorders as well as neurological diseases [80]. The BDNF is a member of the neurotrophin family of proteins. It is involved in the normal development of the peripheral and central nervous system and neuronal survival and synaptic plasticity in the adult brain [81]. Changes in the levels and activities of BDNF have been described in several neurodegenerative disorders like AD and PD [82]. Meanwhile, BDNF also plays a role in psychiatric disorders [83]. Therefore, these three pathways might be involved in the shared pathogenesis of FTD and psychiatric disorders, and future research could attach importance to them.

## Conclusions

By integrating GWAS summary data and the conditional FDR statistical method, we identified selective pleiotropy and novel shared loci between FTD and psychiatric disorders. We further identified shared risk genes *MAPT* and *CADM2* by combining eQTL analysis. MR analysis suggested a nominal causal association between FTD and SCZ. These findings could provide novel insights into the genetic overlap between FTD and psychiatric diseases and help better understand the etiology of FTD.

## Supplementary Information

**Additional file 1: Table S1.** Summary data from all GWAS used in current study. **Table S2.** Risk loci for FTD conditional on psychiatric disorders. **Table S3.** Shared risk loci between FTD subtypes and psychiatric disorders. **Table S4.** Risk loci for FTD subtypes conditional on psychiatric disorders. **Table S5.** eQTL revealing functional effects of shared risk SNPs in human brain tissues. **Table S6.** Enriched pathways from shared risk genes. **Table S7.** Enriched gene ontology from shared risk genes. **Table S8.** Heterogeneity and horizontal pleiotropy analyses between frontotemporal dementia and psychiatric disorders. **Figure S1.** Genetic correlation between subtypes of FTD and psychiatric diseases. **Figure S2.** Conditional quantile-quantile plots of nominal versus empirical -log_10_(P) of each psychiatric disease as a function of significance of association with FTD. **Figure S3.** Fold-enrichment plots of nominal -log_10_(P) of psychiatric diseases as a function of significance of association with FTD. **Figure S4.** Mendelian randomization analysis results between FTD and schizophrenia.

**Additional file 2:** **Supplementary Methods**.

## Data Availability

The GWAS summary statistics used to perform the analyses described in the study were obtained from publicly available published data. The dataset supporting the conclusions of this article is included within the article and its additional files.

## Abbreviations

FTD: Frontotemporal dementia
GWAS: Genome-wide association study
SNP: Single nucleotide polymorphism
LD: Linkage disequilibrium
FDR: False discovery rate
eQTL: Expression quantitative trait locus
GO: Gene ontology
MHC: Major histocompatibility complex
BD: Bipolar disorder
MDD: Major depressive disorder
BDNF: Brain derived neurotrophic factor
ASD: Autism spectrum disorder
ADHD: Attention deficit hyperactivity disorder
AUD: Alcohol use disorder
OCD: Obsessive compulsive disorder
PTSD: Post-traumatic stress disorder
SCZ: Schizophrenia
TS: Tourette’s syndrome
MR: Mendelian randomization
