# Supplementary material for: Shared genetic links between frontotemporal dementia and psychiatric disorders
Source: BMC Med. 2022 May 5;20:131. doi: 10.1186/s12916-022-02335-y (PMC9069762; doi:10.1186/s12916-022-02335-y)
Supplement: Supplementary file 1 — Additional file 1: Table S1. Summary data from all GWAS used in current study. Table S2. Risk loci for FTD conditional on psychiatric disorders. Table S3. Shared risk loci between FTD subtypes and psychiatric disorders. Table S4. Risk loci for FTD subtypes conditional on psychiatric disorders. Table S5. eQTL revealing functional effects of shared risk SNPs in human brain tissues. Table S6. Enriched pathways from shared risk genes. Table S7. Enriched gene ontology from shared risk genes. Table S8. Heterogeneity and horizontal pleiotropy analyses between frontotemporal dementia and psychiatric disorders. Figure S1. Genetic correlation between subtypes of FTD and psychiatric diseases. Figure S2. Conditional quantile-quantile plots of nominal versus empirical -log10(P) of each psychiatric disease as a function of significance of association with FTD. Figure S3. Fold-enrichment plots of nominal -log10(P) of psychiatric diseases as a function of significance of association with FTD. Figure S4. Mendelian randomization analysis results between FTD and schizophrenia. [file 12916_2022_2335_MOESM1_ESM.pdf]

Supplementary tables and figures

Table S1. Summary data of all GWAS used in current study

Disease	Abbr.	Cases	Controls	Ethnics	SNPs	PMID	Authors
frontotemporal dementia	FTD	2,154	4,308	EUR	6,026,384	24943344	Raffaele Ferrari et al.
behavioral variant FTD	bvFTD	1,377	2,754	EUR	6,026,516	24943344	Raffaele Ferrari et al.
semantic dementia	FTD-SD	308	616	EUR	6,026,271	24943344	Raffaele Ferrari et al.
progressive non-fluent aphasia	FTD-PNFA	269	538	EUR	6,026,254	24943344	Raffaele Ferrari et al.
FTD overlapping with motor neuron disease	FTD-MND	200	400	EUR	6,026,165	24943344	Raffaele Ferrari et al.
schizophrenia	SCZ	33,640	43,456	EUR	11,308,216	31740837	Max Lam et al.
attention deficit hyperactivity disorder	ADHD	20,183	35,191	EUR	8,094,094	30478444	Ditte Demontis et al.
autism spectrum disorder	ASD	18,381	27,969	EUR	9,112,386	30804558	Jakob Grove et al.
alcohol use disorders	AUD	121,604	-	EUR	16,213,998	30336701	Sandra Sanchez-Roige et al.
bipolar disorder	BD	14,879	30,992	EUR	6,371,916	31043756	Eli A Stahl et al.
major depressive disorder	MDD	170,756	329,443	EUR	8,483,301	29662059	David M Howard et al.
obsessive-compulsive disorder	OCD	2,688	7,037	EUR	8,409,516	28761083	IOCDF-GC and OCGAS
post-traumatic stress disorder	PTSD	32,428	174,227	EUR	9,766,174	31594949	Caroline M Nievergelt et al.
Tourette’s syndrome	TS	4,819	9,488	EUR	8,265,318	30818990	Dongmei Yu et al.

Abbr., abbreviation; EUR, European; SNPs, number of single nucleotide polymorphism; GWAS, genome-wide association study; PMID, Pubmed ID.

Table S2. Risk loci for FTD conditional on psychiatric disorders

index SNP	Genomic position	Closest gene	FDR value	Associated phenotype	Original FTD P value
rs10863728	1:209056568	LINC01717; LINC01774	0.00597	ASD	5.13E-04
rs10889502	1:65379982	JAK1	0.00464	AUD	3.59E-04
rs12052368	2:16025675	LINC01804; MYCNUT	0.00863	PTSD	7.55E-07
rs79207879	3:139684634	CLSTN2	0.00693	ADHD,BD,PTSD, schizophrenia	5.51E-07
rs9812061	3:85013262	CADM2	0.00777	AUD	4.78E-03
rs3132451	6:31582025	AIF1	0.00699	schizophrenia	8.98E-05
rs3130291	6:32175331	NOTCH4	0.00538	schizophrenia	7.31E-05
rs3117097	6:32358689	HCG23; TSBP1-AS1	0.00003	ADHD,ASD,BD,MDD,OCD, PTSD,schizophrenia,TS	5.54E-08
rs3129953	6:32361821	BTNL2	0.00012	ADHD,ASD,BD,MDD,OCD, PTSD,schizophrenia,TS	5.16E-08
rs9268881	6:32431606	HLA-DRA; HLA-DRB5	1.72E-06	ADHD,ASD,AUD,BD,MDD, OCD,PTSD,schizophrenia,TS	2.39E-10
rs60045856	6:32799845	TAP2	0.00982	schizophrenia	1.43E-04
rs2094494	13:71419022	ATXN8OS; LINC00348	0.00357	ADHD,AUD,BD,MDD,PTS D, schizophrenia	2.65E-07
rs2470183	15:51679797	GLDN	0.00476	schizophrenia	9.46E-07
rs215034	16:15996556	FOPNL; ABCC1	0.00310	AUD	7.30E-04
rs12934137	16:73741667	LINC01568; LOC101928035	0.00537	ADHD,AUD,BD,PTSD, schizophrenia	3.99E-07
rs79724577	17:43463493	MAP3K14	0.00413	AUD	1.68E-04
rs76344126	17:43503284	ARHGAP27	0.00160	ASD,AUD	3.15E-04
rs56314414	17:43536970	PLEKHM1	0.00152	ASD,AUD	3.07E-04
rs12150390	17:43896228	CRHR1;	0.00085	ASD,AUD	2.95E-04

		LINC02210-CRHR1			
rs62054815	17:43923266	SPPL2C	0.00093	AUD	2.75E-04
rs34097347	17:43949448	MAPT-AS1	0.00087	AUD	3.02E-04
rs62063279	17:44036936	MAPT	0.00087	ASD,AUD	2.98E-04
rs62063675	17:44126575	KANSL1	0.00110	AUD	7.53E-04
rs199531	17:44830414	NSF	0.00218	ASD,AUD	1.02E-03
rs199498	17:44865603	WNT3	0.00139	ASD,AUD	8.15E-04
rs6857	19:45392254	NECTIN2	0.00621	AUD	9.26E-07
rs11556505	19:45396144	TOMM40	0.00653	AUD,PTSD	7.54E-07
rs769449	19:45410002	APOE	0.00510	ADHD,AUD,PTSD, schizophrenia	2.99E-07
rs1406857	20:37362432	SLC32A1; ACTR5	0.00421	schizophrenia	4.44E-05

SNP, single nucleotide polymorphism; FDR, false discovery rate; ASD, autism spectrum disorder; AUD, alcohol use disorder; PTSD, post-traumatic stress disorder; ADHD, attention deficit hyperactivity disorder; MDD, major depressive disorder; AUD, alcohol use disorder; OCD, Obsessive compulsive disorder; BD, bipolar disorder; TS, Tourette’s syndrome. Index SNP was the SNP with the lowest FDR value in each locus. The genomic position was on GRCh37. Closet gene was annotated from ANNOVAR.

Table S3. Risk loci for FTD subtypes conditional on psychiatric disorders.

FTD subtype	index SNP	Genomic position	Closest gene	FDR value	Associated phenotype	Original FTD P value
bvFTD	rs74977128	11:87936874	MIR3166;CTSC	0.00262	ADHD	3.06E-08
bvFTD	rs17652337	17:44083323	MAPT	0.00927	AUD	3.29E-03
bvFTD	rs9268887	6:32431833	HLA-DRA;HLA-DRB5	0.00192	SCZ	1.03E-05
FTD_SD	rs7267772	20:21534970	NKX2-2;LINC01727	0.00766	ASD	9.52E-05

SNP, single nucleotide polymorphism; FDR, false discovery rate; ASD, autism spectrum disorder; AUD, alcohol use disorder; ADHD, attention deficit hyperactivity disorder. Index SNP was the SNP with the lowest FDR value in each locus. The genomic position was on GRCh37. Closet gene was annotated from ANNOVAR.

Table S4. Shared risk loci between FTD subtypes and psychiatric disorders.

FTD subtype	index SNP	Genomic position	Closest gene	FDR value	Associated phenotype	Original FTD P value
bvFTD	rs11018787	11:87919202	MIR3166;CTSC	0.00464	ADHD	3.06E-08
bvFTD	rs17652337	17:44083323	MAPT	0.00927	AUD	3.29E-03
bvFTD	rs9268887	6:32431833	HLA-DRA;HLA-DRB5	0.00192	SCZ	1.03E-05
FTD_SD	rs7267772	20:21534970	NKX2-2;LINC01727	0.00766	ASD	9.52E-05

SNP, single nucleotide polymorphism; FDR, false discovery rate; ASD, autism spectrum disorder; AUD, alcohol use disorder; ADHD, attention deficit hyperactivity disorder. Index SNP was the SNP with the lowest FDR value in each locus. The genomic position was on GRCh37. Closet gene was annotated from ANNOVAR.

Table S5. eQTL revealing functional effects of shared risk SNPs in human brain tissues

Target protein	eQTL in Braineac			eQTL in GTEx		
	Associated SNP	Closest gene	tissues	Associated SNP	Closest gene	tissues
MAPT	rs112454267	MAPT	FCTX,TCTX	rs10445369	MAPT	Brain_Cerebellum
LRRC37A2	rs112454267	LRRC37A2	CRBL,FCTX, HIPP,MEDU, OCTX,PUTM, SNIG,TCTX, THAL,WHMT	rs62062322	LRRC37A2	Cerebellar_Hemisphere, Spinal_cord_cervical_c-1, Nucleus accumbens basal ganglia, Anterior_cingulate_cortex_BA24, Substantia nigra, Cortex, Frontal_Cortex_BA9, Cerebellum, Amygdala, Caudate_basal_ganglia, Putamen basal_ganglia, Hippocampus, Hypothalamus
CADM2	rs13062439	CADM2	WHMT	rs11719276	CADM2	Spinal_cord_cervical_c-1

SNP, single nucleotide polymorphism; FDR, false discovery rate; FCTX, frontal cortex; TCTX, temporal cortex; CRBL, cerebellar cortex; HIPP, hippocampus; MEDU, medulla; OCTX, occipital cortex; PUTM, putamen; SNIG, substantia nigra; THAL, thalamus; WHMT, intralobular white matter. Target protein is the protein of which expression was influenced by shared risk SNPs. Closest gene was annotated using ANNOVAR for associated SNP.

Table S6. Enriched pathways from shared risk genes

pathway name	P value	FDR adjusted P value	pathway source
IL-5 signaling pathway	0.00056	0.00556	Wikipathways
IL-2 signaling pathway	0.000617	0.00556	Wikipathways
Synaptic Vesicle Pathway	0.00091	0.00614	Wikipathways
Transmission across Chemical Synapses	0.00122	0.0066	Reactome
Synaptic vesicle cycle - Homo sapiens (human)	0.00212	0.00952	KEGG
GABAergic synapse - Homo sapiens (human)	0.00274	0.00967	KEGG
Class B/2 (Secretin family receptors)	0.00287	0.00967	Reactome
Neuronal System	0.00443	0.0124	Reactome
ESC Pluripotency Pathways	0.00461	0.0124	Wikipathways
Signaling pathways regulating pluripotency of stem cells - Homo sapiens (human)	0.00692	0.0158	KEGG
Brain-derived neurotrophic factor (BDNF) signaling pathway	0.00702	0.0158	Wikipathways
Cushing syndrome - Homo sapiens (human)	0.00809	0.0168	KEGG

Table S7. Enriched gene ontology from shared risk genes

GO ID		P value	FDR adjusted P value
GO:0098805	whole membrane	0.000794	0.0175
GO:0019904	protein domain specific binding	0.00118	0.0189
GO:0043005	neuron projection	0.00171	0.024
GO:0098852	lytic vacuole membrane	0.00229	0.024
GO:0005765	lysosomal membrane	0.00229	0.00687
GO:0061387	regulation of extent of cell growth	0.00245	0.0433
GO:0015238	drug transmembrane transporter activity	0.00274	0.0219
GO:0048675	axon extension	0.00294	0.0433
GO:0017124	SH3 domain binding	0.00369	0.03
GO:0031410	cytoplasmic vesicle	0.00444	0.031
GO:0019903	protein phosphatase binding	0.0049	0.0245
GO:1903825	organic acid transmembrane transport	0.00536	0.0433
GO:0030307	positive regulation of cell growth	0.00556	0.0433
GO:0048639	positive regulation of developmental growth	0.0057	0.0433
GO:0046943	carboxylic acid transmembrane transporter activity	0.00605	0.03
GO:0005342	organic acid transmembrane transporter activity	0.00612	0.0326
GO:0021953	central nervous system neuron differentiation	0.00723	0.0458
GO:0019902	phosphatase binding	0.00819	0.03
GO:0030425	dendrite	0.00878	0.0317
GO:0097447	dendritic tree	0.00886	0.0317
GO:0030424	axon	0.00906	0.0317

GO, Gene Ontology; FDR, false discovery rate.

Table S8. Heterogeneity and horizontal pleiotropy analyses between frontotemporal dementia and psychiatric disorders.

exposure trait	outcome trait	Heterogeneity			Horizontal pleiotropy			MR-PRESSO P value	Beta
		IVW Q	IVW Q df	IVW P value	Egger intercept	SE	P value		
ADHD	FTD	6.47	5	0.26	-0.18	0.10	0.13	0.29	0.85
ASD		8.59	8	0.38	0.05	0.11	0.67	0.37	0.80
AUD		12.39	7	0.09	0.03	0.06	0.58	0.13	1.00
BD		13.05	13	0.29	-0.01	0.10	0.91	0.31	0.44
MDD		49.10	41	0.18	-0.04	0.04	0.32	0.17	0.96
OCD		3.40	3	0.33	0.12	0.07	0.26	0.33	0.55
PTSD		3.74	3	0.29	0.04	0.21	0.87	0.33	1.61
SCZ		70.22	68	0.40	-0.04	0.02	0.07	0.42	0.34
TS		2.26	3	0.52	-0.16	0.18	0.46	0.52	0.73
ADHD	bvFTD	5.89	5	0.32	-0.25	0.12	0.10	0.36	1.01
ASD		9.23	8	0.32	0.11	0.14	0.45	0.35	0.96
AUD		10.13	7	0.18	0.00	0.07	0.96	0.25	1.17
BD		9.53	11	0.57	0.08	0.11	0.49	0.60	0.54
MDD		48.14	41	0.21	-0.04	0.05	0.46	0.18	1.12
OCD		6.75	3	0.08	0.14	0.13	0.40	0.09	0.67
PTSD		5.00	3	0.17	0.28	0.28	0.62	0.22	1.75
SCZ		65.09	68	0.58	-0.01	0.03	0.67	0.59	0.39
TS		0.87	3	0.83	-0.12	0.22	0.64	0.86	0.88
ADHD	FTD_MND	8.16	5	0.15	-0.81	0.31	0.06	0.14	1.70
ASD		9.51	8	0.30	0.06	0.39	0.88	0.29	1.66
AUD		4.36	7	0.74	-0.02	0.14	0.88	0.76	1.81
BD		13.81	11	0.24	-0.69	0.28	0.03	0.27	1.16
MDD		50.06	41	0.16	-0.04	0.14	0.76	0.15	1.78
OCD		4.59	3	0.20	0.09	0.37	0.82	0.24	1.35
PTSD		0.31	3	0.96	-0.35	0.68	0.66	0.97	2.04
SCZ		85.66	68	0.07	-0.14	0.09	0.09	0.08	0.94
TS		3.17	3	0.37	-0.04	0.72	0.96	0.40	1.59
ADHD	FTD_PNFA	7.47	5	0.19	0.38	0.33	0.31	0.17	1.61

ASD		7.89	8	0.44	0.34	0.29	0.29	0.46	1.56
AUD		16.27	7	0.02	0.01	0.21	0.96	0.05	1.74
BD		15.27	11	0.17	-0.29	0.31	0.37	0.16	1.05
MDD		35.10	41	0.73	-0.02	0.11	0.84	0.73	1.70
OCD		4.57	3	0.21	0.26	0.27	0.43	0.20	1.24
PTSD		4.10	3	0.25	-0.05	0.52	0.94	0.20	2.02
SCZ		64.00	68	0.62	-0.18	0.07	0.01	0.61	0.84
TS		1.82	3	0.61	-0.29	0.52	0.63	0.58	1.48
ADHD		3.78	5	0.58	0.09	0.25	0.75	0.54	1.57
ASD		13.29	8	0.10	-0.45	0.32	0.20	0.14	1.52
AUD		9.41	7	0.22	0.24	0.12	0.08	0.21	1.70
BD		12.61	11	0.32	0.28	0.24	0.28	0.34	1.00
MDD	FTD_SD	45.32	41	0.30	-0.08	0.10	0.42	0.31	1.66
OCD		0.77	3	0.86	-0.14	0.20	0.56	0.83	1.19
PTSD		0.62	3	0.89	-0.20	0.42	0.69	0.86	2.01
SCZ		79.11	68	0.17	-0.01	0.07	0.88	0.17	0.80
TS		1.10	3	0.78	-0.32	0.47	0.57	0.79	1.43
	ADHD	3.69	6	0.72	0.03	0.02	0.23	0.74	0.14
	ASD	12.40	6	0.05	0.00	0.04	0.90	0.08	0.15
	AUD	9.57	6	0.14	0.00	0.00	0.88	0.17	0.11
	BD	8.84	4	0.07	0.05	0.04	0.30	0.11	0.19
FTD	MDD	1.11	5	0.95	0.00	0.01	0.97	0.95	0.06
	OCD	7.74	5	0.17	-0.09	0.07	0.30	0.19	0.41
	PTSD	7.46	6	0.28	-0.02	0.03	0.58	0.26	0.12
	SCZ	6.60	5	0.25	0.00	0.02	0.91	0.30	0.14
	TS	3.73	5	0.59	-0.08	0.05	0.18	0.66	0.33

IVW, Inverse variance weighted; Q, Cochran's Q test estimate; df, Cochran's Q test degrees of freedom; SE, standard error; ASD, autism spectrum disorder; AUD, alcohol use disorder; PTSD, post-traumatic stress disorder; ADHD, attention deficit hyperactivity disorder; MDD, major depressive disorder; AUD, alcohol use disorder; OCD, Obsessive compulsive disorder; BD, bipolar disorder; SCZ, schizophrenia; TS, Tourette's syndrome; FTD, frontotemporal dementia; bvFTD, behavioral variant FTD; SD, semantic dementia; PNFA, progressive non-fluent aphasia; MND, motor neuron disease. Beta means the effect size can be detected with the power of 0.8 given the sample size, proportion of cases and variance explained by instrumental variables.

Fig. S1: Genetic correlation between subtypes of FTD and psychiatric diseases.

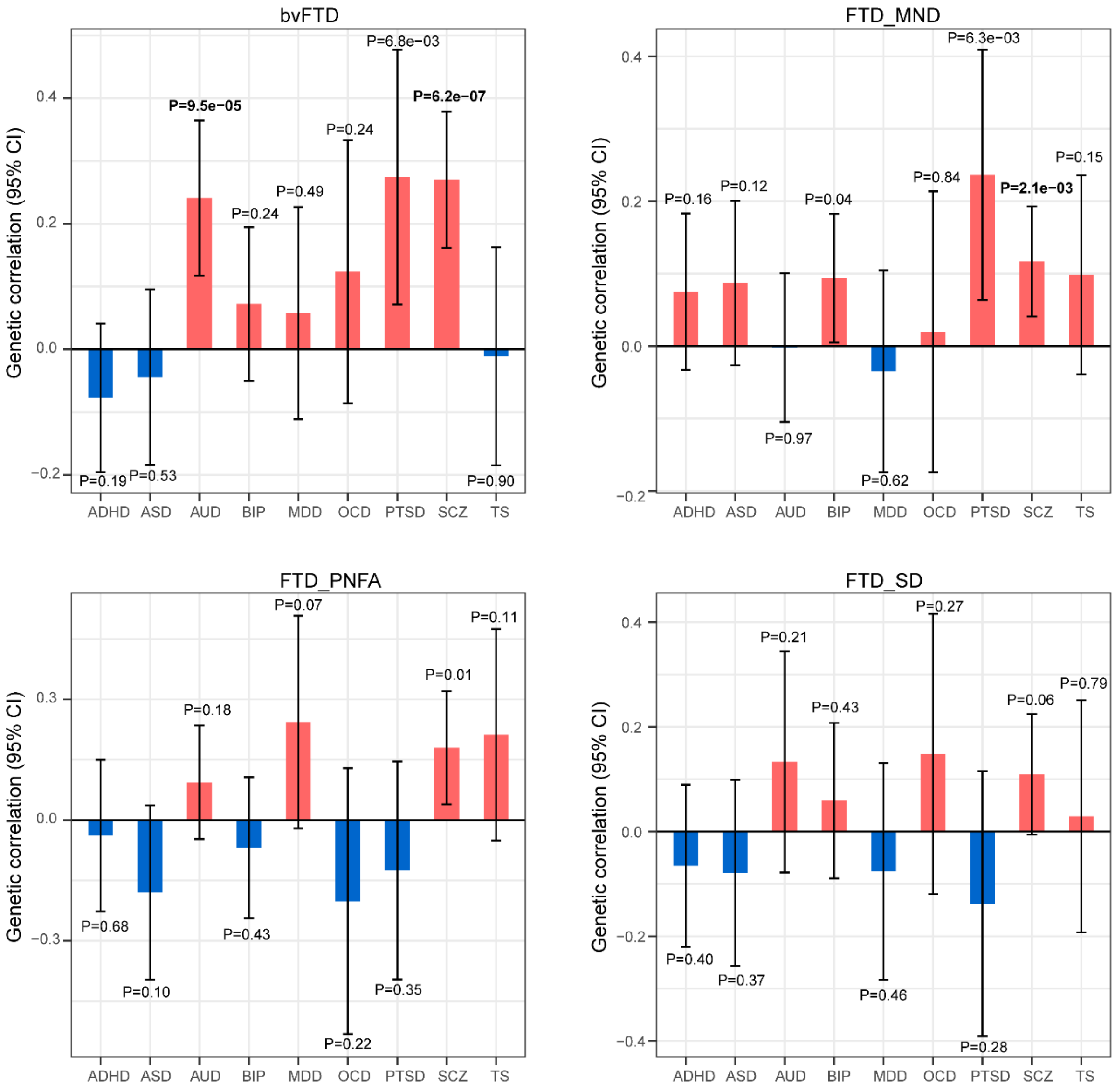

Error bars indicate 95% confidence intervals. Red color indicates positive correlation, while blue color indicates negative correlation. Bold P value denotes significance after the Bonferroni correction.

Fig. S2. Conditional quantile-quantile plots of nominal versus empirical -log10(P) of each psychiatric disease as a function of significance of association with FTD.

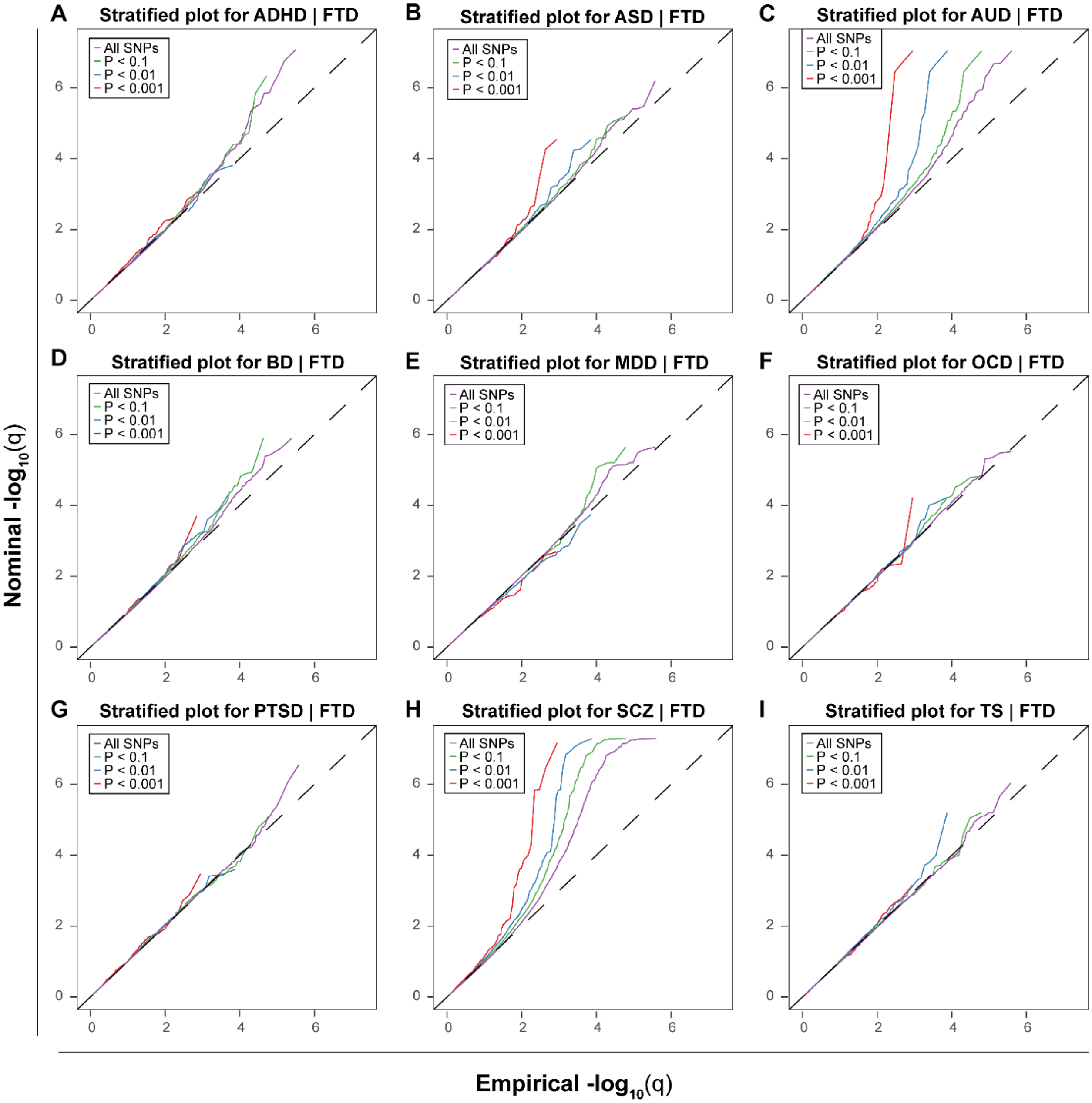

Fig. S3. Fold-enrichment plots of nominal -log10(P) of psychiatric diseases as a function of significance of association with FTD.

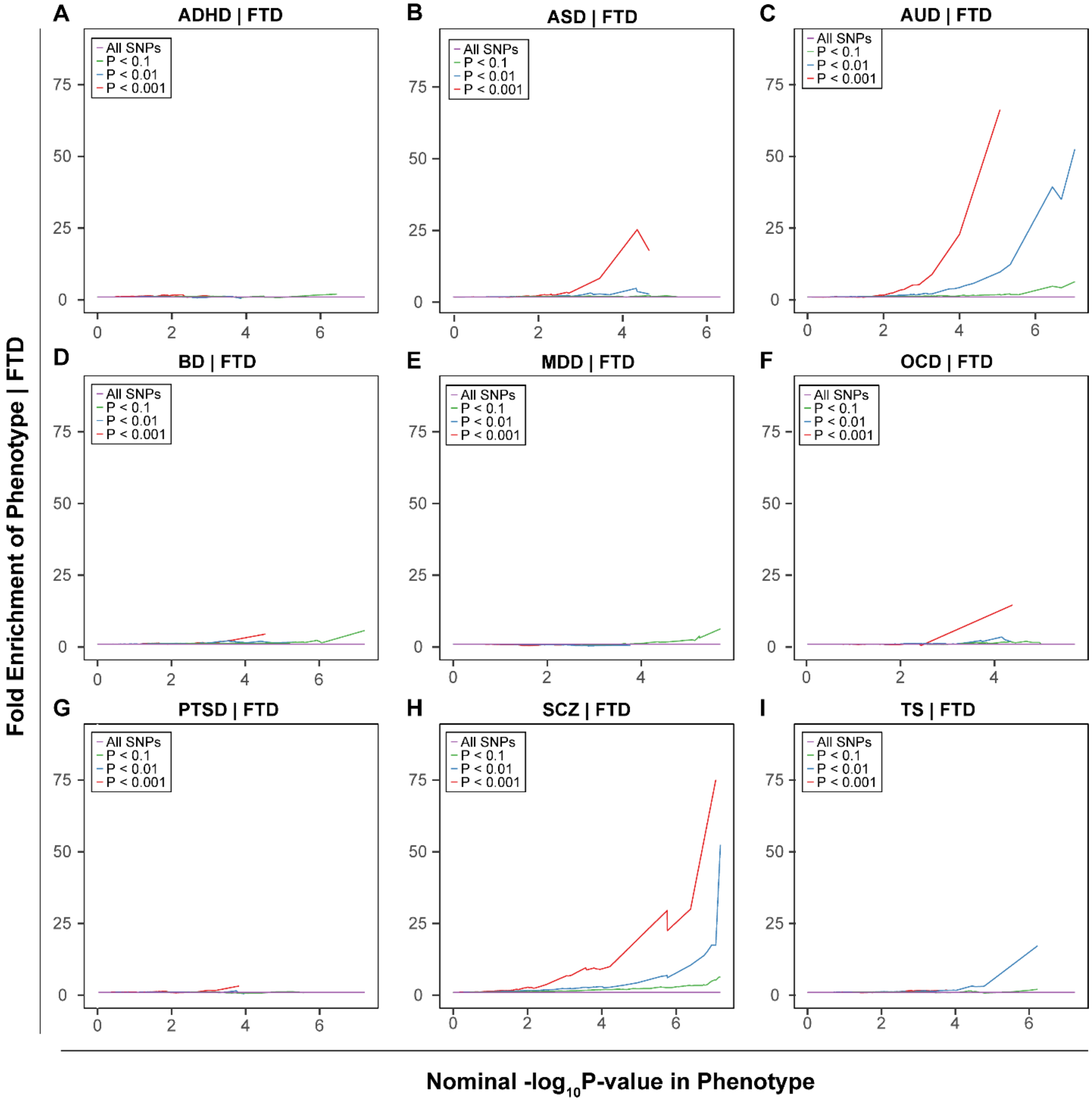

Fig. S4: Mendelian randomization analysis results between FTD and schizophrenia.

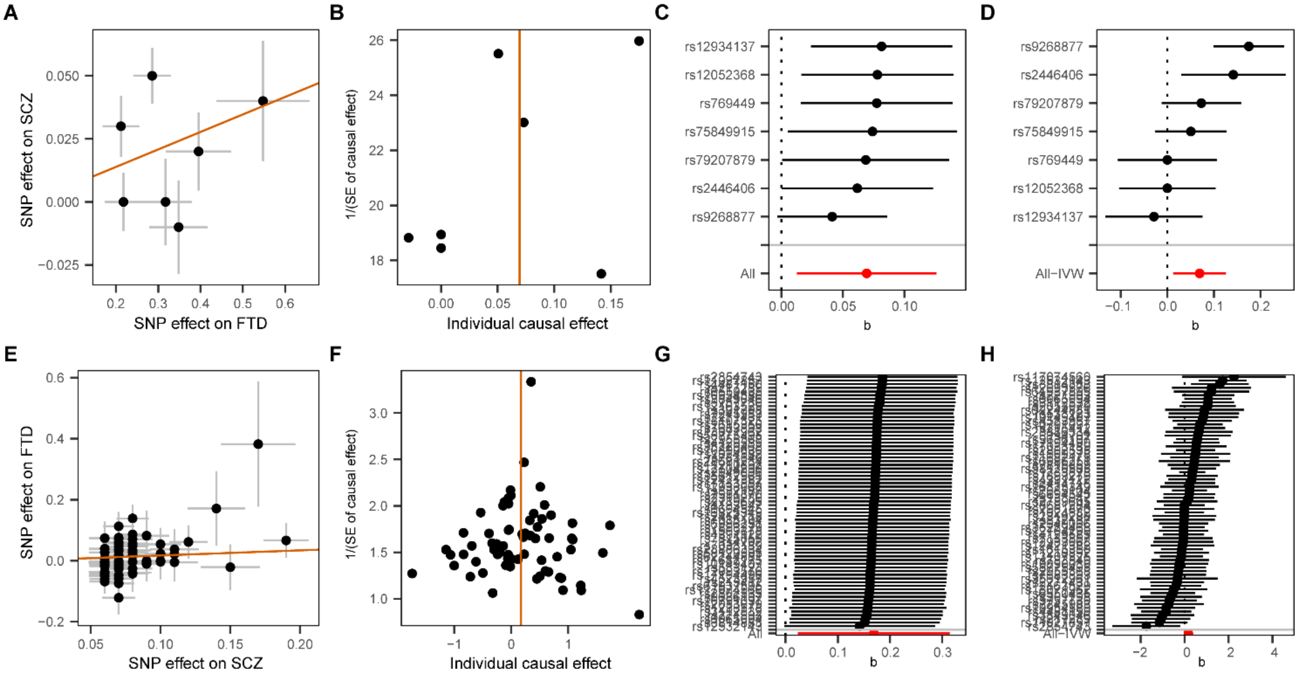

FTD, frontotemporal dementia; SCZ, schizophrenia. (**A, B, C, D**) Mendelian randomization analysis results with FTD as exposure and SCZ as outcome. (**E, F, G, H**) Mendelian randomization analysis results with SCZ as exposure and FTD as outcome. (**A, E**): Scatter plot of single nucleotide polymorphism (SNP) effects on FTD and schizophrenia. The slope of fitted lines represents the estimated effect. (**B, F**): Funnel plot shows the estimation using the inverse of the standard error of the causal estimate with each individual SNP as a tool. The vertical line represents the estimated causal effect obtained using IVW method. (**C, G**): Forest plot of the results of the leave-one-out sensitivity analysis, where each SNP in the instrument was iteratively removed from the instrument variables. (**D, H**): Forest plot of the effect of each SNP in the MR analysis.
