# Supplementary material for: Shared genetic links between frontotemporal dementia and psychiatric disorders
Source: BMC Med. 2022 May 5;20:131. doi: 10.1186/s12916-022-02335-y (PMC9069762; doi:10.1186/s12916-022-02335-y)
Supplement: Supplementary file 2 — Additional file 2: Supplementary Methods. [file 12916_2022_2335_MOESM2_ESM.pdf]

Supplementary Methods

Genomic Control

The empirical null distribution in genome-wide association studies (GWAS) is sometimes inflated due to population stratification, cryptic relatedness, or deflated due to over-correction of test statistics. We applied a genomic control method leveraging only intergenic SNPs, which are likely depleted for true associations and could provide a robust estimate of true null effects. First, we annotated the SNPs to genic (5'UTR, exon, intron, 3'UTR) and intergenic regions using ANNOVAR. We converted all P values to Z scores and then estimated the genomic inflation factor λ_{GC} for each trait. λ_{GC} was calculated as the median Z score squared divided by the expected median of a chi-square distribution with one degree of freedom. Then all P values were divided by λ_{GC} for genomic control adjustment. After this, we pruned the SNPs by removing SNPs in linkage disequilibrium (LD) ($r^2>0.2$ within 250kb) based on 1000 Genomes Project LD structure using plink clump functionality.

QQ plots

Quantile-quantile plots compare a nominal probability distribution against an empirical distribution, and leftward deflection of the observed distribution reflects enrichment of low P values. Specifically, we computed the empirical cumulative distribution of nominal P values of FTD for all SNPs and subsets of SNPs with significance level below the indicated cutoffs in each psychiatric disease ($-\log_{10}(P) \geq 0$, $-\log_{10}(P) \geq 1$, $-\log_{10}(P) \geq 2$, $-\log_{10}(P) \geq 3$), corresponding to $P \leq 1$, $P \leq 0.1$, $P \leq 0.01$, $P \leq 0.001$). To assess for polygenic effects below the standard GWAS significance threshold, we focused on SNPs with nominal $-\log_{10}(P) < 7.3$ (corresponding to P > 5E-08).

Fold enrichment plots

We built fold enrichment plots to quantitatively assess the genetic enrichment between two phenotypes. For a given associated phenotype, enrichment for pleiotropy is present if the degree of deflection from the expected null line is dependent on SNP associations with the second phenotype. Specifically, we computed the empirical cumulative distribution of nominal P values for FTD for all SNPs and for SNPs with significance levels below the indicated cut-offs for each psychiatric disorder ($-\log_{10}(P) \geq 0$, $-\log_{10}(P) \geq 1$, $-\log_{10}(P) \geq 2$, $-\log_{10}(P) \geq 3$ corresponding to $P \leqslant 1$, $P \leqslant 0.1$, $P \leqslant 0.01$, $P \leqslant 0.001$ respectively). The nominal P values ($-\log_{10}(P)$) are plotted on the X-axis, and fold enrichment in FTD as a function of each psychiatric disorder is plotted on the Y-axis. To assess for polygenic effects below the standard GWAS significance threshold, we focused on SNPs with nominal $-\log_{10}(P) < 7.3$ (corresponding to $P > 5E-08$).

Conditional/Conjunctional FDR statistics

Details of the conditional/conjunctional FDR statistics have been described in the original publication introducing the cFDR method. Briefly, the FDR method is based on Bayesian statistics, and the conditional FDR is the probability of the SNP being null given its P value is as small as or smaller than observed. The conjunctional FDR is an extension of the conditional FDR and is defined as the maximum of the two conditional FDR statistics for a specific SNP. We defined the conjunctional statistics (denoted as $FDR_{Trait1\ \&\ Trait2}$) as the maximum of the conditional FDR in both directions, i.e.

$$FDR_{Trait1\ \&\ Trait2} = \max(FDR_{Trait1\ |\ Trait2}, FDR_{Trait2\ |\ Trait1})$$

based on the combination of P value for the SNP in FTD and each psychiatric disease. The conjunctional statistic allows for the identification of SNPs that are associated with both phenotypes, which minimizes the effect of a single phenotype driving the common association signal.
